# A series of unfortunate bladder events: An illustrative case series of a diverse cohort of bladder perforations

**DOI:** 10.1016/j.radcr.2022.08.010

**Published:** 2022-09-06

**Authors:** Steven Anderson, Kenneth Patterson, Niall F. Davis, Mark R. Quinlan

**Affiliations:** a Department of Urology, Beaumont Hospital, Beaumont Rd, Dublin, Ireland; b Department of Surgical Affairs, RCSI University of Medicine and Health Sciences, Dublin, Ireland

**Keywords:** Bladder perforation, Bladder injury, Intraperitoneal, Extraperitoneal, Trauma

## Abstract

Bladder perforation is a potentially life-threatening condition, typically occurring after genitourinary trauma. The vast majority of cases are secondary to blunt abdominal trauma resulting in pelvic fractures, with motor vehicle accidents the commonest cause. There are however a wide range of underlying causes, including iatrogenic injuries and spontaneous perforations. This case series of 4 unusual cases of bladder perforations presenting to a single center under the same consultant within a 3-month period aims to highlight the diverse nature of patients who can present with bladder perforations and the different management options available.

## Introduction

Bladder injuries are not uncommon and occur in up to 10% of abdominal trauma [1]. They encompass a wide array of clinical presentations and can be life-threatening in severe cases. The American Association of Surgical Trauma has developed a grading system to categorize the severity of bladder injuries, though perforations are more commonly and more usefully classified as extraperitoneal, intraperitoneal or combined [2]. The commonest cause of bladder perforation is blunt abdominal trauma and up to 90% of cases are associated with pelvic fractures [3]. Approximately 60% of bladder perforations are extraperitoneal which are typically managed conservatively with bladder drainage and supportive measures [4].

The majority of patients with significant bladder injuries present with visible hematuria, blood per urethra (PU), and/or lower abdominal pain [5]. Furthermore, an intraperitoneal bladder perforation (IBP) should be suspected in trauma patients presenting with abdominal pain with distension and an inability to void.

The preferred method of diagnosis of a bladder perforation has historically been via stress retrograde cystography including post-void images [6]. However, modern trauma series protocols and advancements in computed tomography (CT) have resulted in a decreased use of conventional cystography in favor of CT cystography [7]. There is however no “one size fits all” approach to the investigation, diagnosis and management of bladder perforations, as we aim to illustrate in our case series of diverse patients.

In this case series, we present a single surgeon's experience of 4 patients presenting to our institution with bladder perforations in just a 3-month period. The electronic records of these patients were retrospectively reviewed and the relevant clinicopathological and radiological data were collected. Informed consent was obtained from all patients. This study was granted institutional review board exemption from the Beaumont Hospital ethics committee.

## Case presentations

### Case 1

**Fig. 1:**
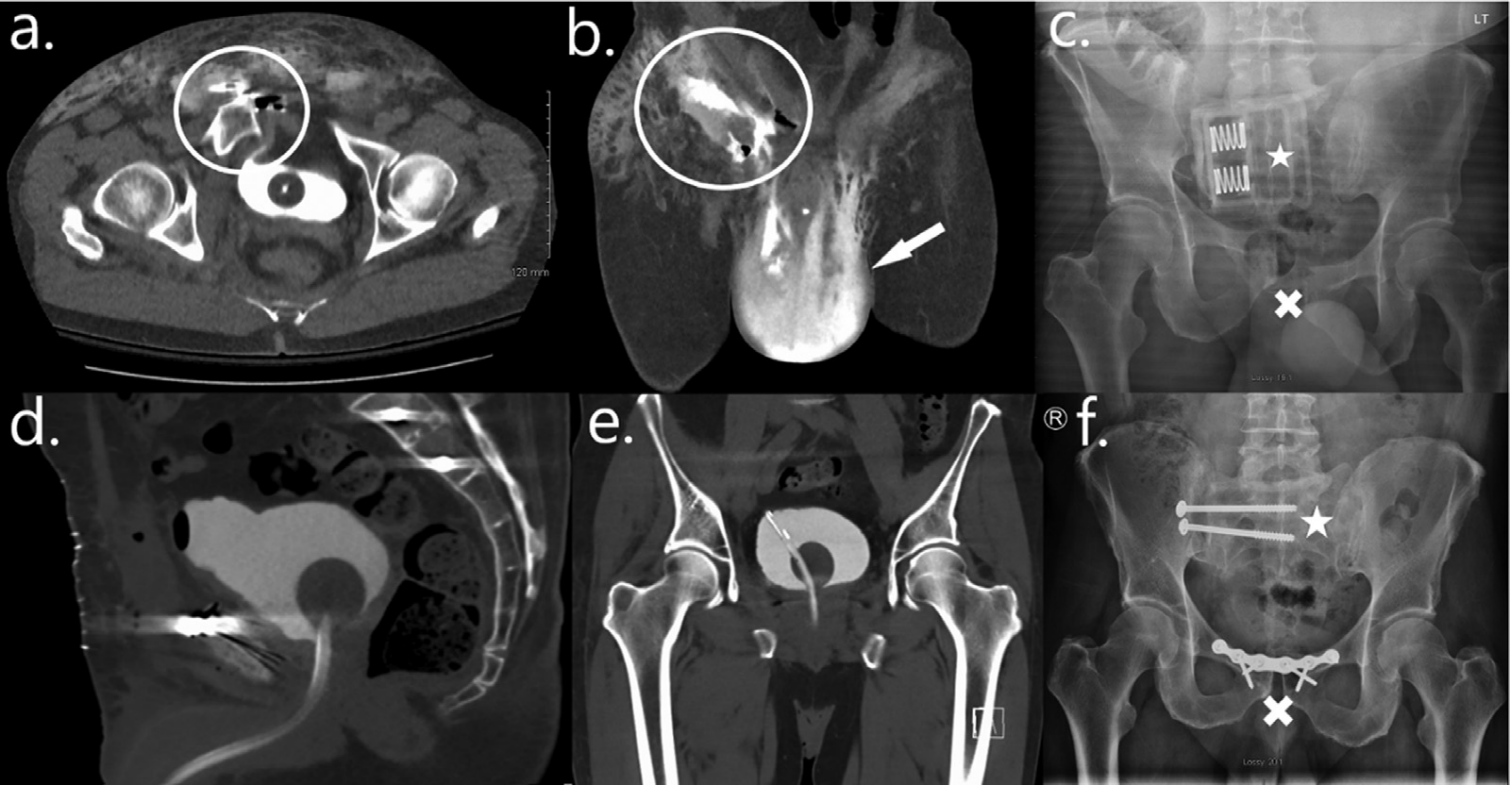
Case 1—Extraperitoneal bladder perforation: Axial (A) and coronal (B) images of a CT urogram demonstrating contrast extravasation (circle) and pooling in the extraperitoneal space with associated inflammation as well as a large collection of contrast within the scrotum (arrow). Postoperative images from a CT cystogram (images D and E) show no residual extravasation. A preoperative pelvic X-ray (C) demonstrates a pelvic fracture (cross) stabilized with a pelvic binder (star). A postoperative pelvic X-ray (F) illustrates the pelvic fracture repair (star and cross).

Case 1 is of a 60-year-old male who presented with a large extraperitoneal bladder perforation sustained following a fall from a height. He was initially brought in by ambulance to his local hospital after he had sleep-walked through a second-floor window, sustaining a significant pelvic fracture. Following initial resuscitation in the emergency department (ED), he underwent a trauma protocol CT with a delayed urographic phase. This demonstrated a large anterior bladder wall defect with active contrast extravasation into his extraperitoneal space, tracking down into his scrotum (Fig. 1). His pelvic fracture was stabilized with a pelvic binder. Upon transfer to our institution, he was found to have marked swelling of his scrotum, which was tense, and expanding erythema of the anterior aspects of his upper thighs and lower abdominal wall. He underwent an emergency laparotomy.

Intraoperatively, he was found to have an 8 cm defect in the anterior wall of his bladder. This was repaired in 2 layers with absorbable sutures. A leak test was performed at the end of the case which was negative. A surgical drain and urethral catheter were left in situ. The drain was removed after 3 days and he was then transferred to the national pelvic/acetabular orthopedic tertiary referral center for definitive repair of his pelvic fracture. He underwent a successful trial without catheter (TWOC) there following a CT cystogram which was performed 10 days after his initial bladder repair (Fig. 1).

### Case 2

**Fig. 2:**
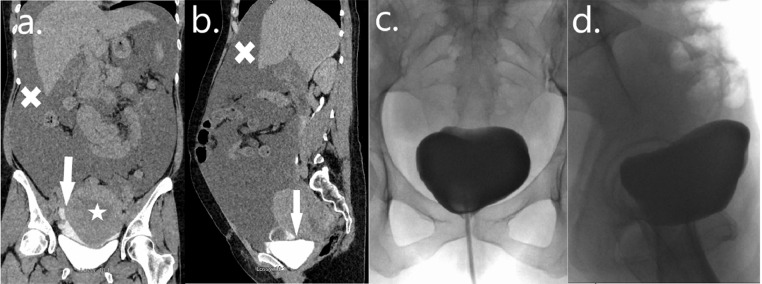
Case 2—Iatrogenic intraperitoneal bladder perforation: Coronal (A) and sagittal (B) images from a CT urogram demonstrating contrast extravasation (arrows) with a large volume of intraperitoneal-free fluid (crosses) and a thickened postpartum uterus (star). Anterior-posterior (C) and lateral (D) images of a retrograde stress cystogram confirming no residual urine leak following conservative management with an indwelling urethral catheter.

Case 2 is of a 38-year-old female who presented to her local hospital's ED with nausea, vomiting, abdominal pain, and visible hematuria 7 days following an elective caesarean section (CS) there. She was found to have a distended abdomen without an obviously palpable bladder. Blood results demonstrated an acute kidney injury (serum creatinine 135 µmol/L) and a Hb of 11g/dL. She was catheterized and resuscitated with intravenous fluids. She underwent a contrast-enhanced CT which showed a thickened uterus with a small hematoma and a trace of intra-abdominal free fluid only, all of which were in keeping with postoperative changes. Her serum creatinine improved initially following rehydration, however, following removal of her urethral catheter her creatinine level increased and she began to leak fluid through her CS wound. She thus underwent a follow-up CT scan on day 10 post-CS with a dedicated urographic phase which demonstrated a large volume of intraperitoneal-free fluid and a suggestion of a small defect in the dome of her bladder (Fig. 2).

Following consultation with ourselves, a large-bore silicone urethral catheter was inserted, intravenous antibiotics were commenced and she was transferred to our institution. Her vital signs were within normal limits, she was clinically well and her distension had improved significantly following catheterization so a decision was made to insert a percutaneous drain in interventional radiology and to treat her conservatively. This drained large volumes initially with a creatinine sample confirming it to be urine. She remained clinically well and her serum creatinine normalized quickly. Her drain output tapered steadily during the first few days of her admission and it was then removed. She was discharged home on oral antibiotics after her drain was removed but with her urethral catheter still in situ and she returned for a stress retrograde cystogram 3 weeks following her transfer to our institution (Fig. 2). This confirmed no residual urine leak and she underwent a successful TWOC later that day.

### Case 3

Case 3 is of an 18-year-old female who presented to our ED with abdominal pain and distension. She had a background history of spina bifida and mobilized with the aid of a wheelchair. She had previously undergone insertion of a ventriculoperitoneal shunt in early childhood, followed by an augmentation cystoplasty and mitrofanoff procedure, through which she performed clean intermittent self-catheterization. On presentation to the ED, she was found to have generalized and significant abdominal tenderness but was clinically quite well. Her blood results on presentation were all within normal limits, however, due the severity of her pain and her background history, she underwent a CT scan following initial fluid resuscitation and insertion of an indwelling urethral catheter via her mitrofanoff.

**Fig. 3:**
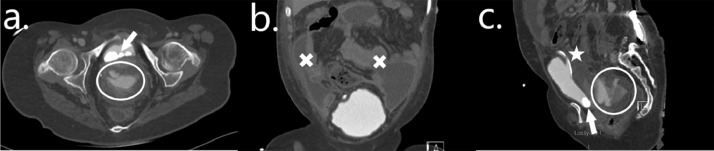
Case 3—Spontaneous intraperitoneal bladder perforation: Axial (A), coronal (B), and sagittal (C) images of a CT urogram. Active contrast extravasation can be seen (circles) with a large volume of intraperitoneal fluid (crosses). Bladder stones (arrows) can be seen within the augmented bladder with a suprapubic catheter (star) in situ.

Her CT revealed multiple stones within her bladder and delayed urographic images confirmed contrast extravasation in keeping with an IBP (Fig. 3).

Given that her pain improved significantly following mitrofanoff catheterization and given that she was apyrexial and clinically well, a decision was made to manage her conservatively with intravenous antibiotics, an indwelling mitrofanoff catheter and placement of a large bore urethral catheter. She remained hemodynamically stable throughout her admission and was discharged home 5 days later with her urethral catheter on free drainage and her mitrofanoff catheter spigotted. She returned for a stress cytogram after 2 weeks which showed no ongoing leak, following which she returned to performing clean intermittent self-catheterization with removal of both catheters. She underwent a successful cystolitholapaxy with complete clearance of her bladder stones 6 weeks after her initial presentation.

### Case 4

**Fig. 4:**
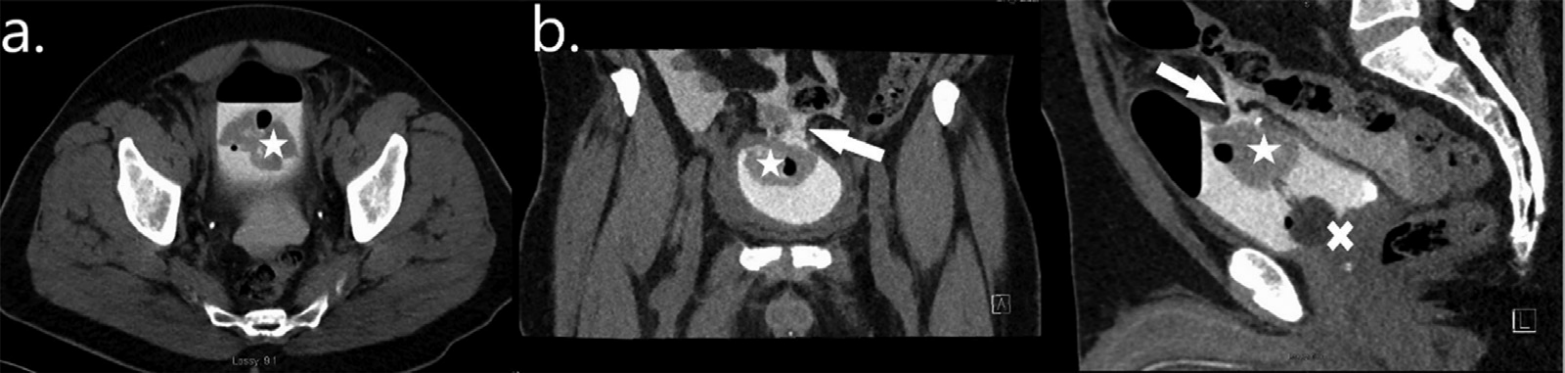
Case 4—Intraperitoneal bladder perforation following a minor fall: Axial (A), coronal (B), and sagittal (C) images from a CT cystogram demonstrating active contrast extravasation (arrows) with a large volume of clot (stars) within the bladder and a urethral catheter (cross) in situ.

Case 4 is of a 59-year-old male who presented to the ED with severe abdominal pain. He suffered a minor and low impact fall from standing the previous night after moderate alcohol consumption. He was hemodynamically stable on presentation but unable to pass urine with blood PU. Examination revealed an exquisitely tender abdomen. A bedside focused assessment with sonography for trauma scan in ED revealed intra-abdominal free fluid. He underwent an urgent CT scan which suggested an IBP, which was then confirmed on a subsequent CT cystogram (Fig. 4). He underwent an emergency laparotomy at which time a large defect in the dome of his bladder was identified with associated perivesical hematoma. The hematoma was manually evacuated; his bladder was washed out and repaired in 2 layers with absorbable sutures. A leak test was unremarkable. An intra-abdominal drain and indwelling urethral catheter were left in situ.

His abdominal drain was removed 3 days postoperatively and he was discharged home on day 8 with his catheter in situ. He returned for a retrograde stress cystogram after 2 weeks which confirmed no ongoing urine leak (Fig. 4), and he underwent a successful TWOC that day.

## Discussion

The description of bladder perforations is often simplified as either extraperitoneal or intraperitoneal, as extraperitoneal perforations can generally be managed conservatively, while IBP typically require surgical repair [1]. Extraperitoneal perforations seen secondary to pelvic fractures sustained from high velocity injuries, such as the injury seen in case one, are the commonest cause of bladder perforations. There are however, to the authors’ knowledge, no other reported cases of such an injury being sustained due to the patient sleep-walking out of a window. Historically, extraperitoneal perforations have been managed with prompt bladder catheterization and supportive measures. Patients suffering from ongoing urine leak despite catheterization have an increased risk of prolonged hospital stays and increased complication rates [8]. Kotkin et al. reported their initial experience of 36 cases of extraperitoneal perforations and although 74% had spontaneous healing within 2 weeks, 26% had severe complications [9]. Johnsen et al. reported a larger series of 80 cases of extraperitoneal perforations and compared outcomes for those managed with catheterization alone to those managed with early surgical repair. Although they found no overall difference in complication rates or intensive care unit stays between the 2 groups, on subgroup analysis they found that patients undergoing major surgical repair for other injuries who did not undergo simultaneous bladder repair had a significantly higher complication rate, longer intensive care unit stay (9.0 vs 4.0 days, *P* = .02) and overall hospital stay (18.0 vs 10.6 days, *P* = .02) compared to those undergoing simultaneous bladder repair [10].

Patient one from our series did not undergo simultaneous bladder and pelvic fracture repair as the severity of his pelvic fracture necessitated transfer to a national pelvic and acetabular orthopedic tertiary referral center for definitive repair. The extent of his urine leak was so significant that the patient had developed significant cellulitis in his upper thighs and abdomen and developed large and tense bilateral scrotal collections due to the extravasated urine before he underwent his laparotomy. In addition, the authors felt that delaying his bladder repair would increase his long-term risk of fistula development and increase the risk of infection at the time of his pelvic fracture repair.

Although iatrogenic injury only accounts for a small percentage of bladder perforations, the bladder is the urological organ most frequently affected by iatrogenic injury [11]. Internal iatrogenic bladder injuries are most commonly seen during transurethral resection of bladder tumors and are usually extraperitoneal in nature, with a reported need for intervention of less than 0.5% [12]. External injuries are most frequently encountered following gynecological or obstetric procedures [11]. Although the incidence of bladder injury following CS has been reported to be as high as 0.9%, delayed presentations of IBP are very rare, with very few reported cases in the literature [13]. IBP following CS are usually managed with early surgical repair, however in our case the patient had stabilized and had no residual pain following initial abdominal and bladder drainage. Aghaways et al. report a similar case of a patient presenting 11 days following CS with an IBP who was managed successfully with insertion of a percutaneous drain and urethral catheter [13].

The standard of care for managing IBP remains urgent surgical repair in order to reduce the risk of peritonitis, sepsis and other sequelae of intraperitoneal extravasation of urine [12]. There is however, a growing body of evidence to support the non-operative management of intraperitoneal perforations in select cases in both the adult and pediatric populations [14,15]. Although the majority of evidence for conservative management is derived from isolated case reports, Lee et al. reported their outcomes of 13 patients with IBP following transurethral resection of bladder tumors who were successfully managed nonoperatively and found no difference in oncological outcomes compared to a matched cohort during their study follow-up period [16]. Extrapolating from their data, and from other reported cases, it is the authors’ opinion that cases such as cases 2 and 3 from our series can be successfully managed with conservative measures and close observation, presuming of course that the patient is clinically well.

Bladder perforations can also occur following minor trauma, often related to alcohol intoxication, and often with a full bladder. Case 4 from our series is such an example and illustrates how even a minor fall can result in a significant bladder injury requiring urgent exploration. Similar cases have been previously reported and there is even a reported case of bladder perforation secondary to a minor fall while dancing [17,18].

Spontaneous bladder perforations (SBP) in the absence of any form of trauma are very rare. Zhang et al. performed a review of the literature and identified 713 cases of SBP, with a reported incidence of 1/126,000. The commonest causes for SBP were alcohol intoxication (39.2%) and lower urinary tract obstruction (18.3%) [18]. In their review, only 0.7% (n = 5) of SBP cases had a previous augmentation enterocystoplasty as a predisposing risk factor for perforation; however, it is unclear if all included cases in their review presented with SBP in the absence of any form of abdominal or pelvic trauma, as seen in case 3 of our series.

## Conclusion

Although this is a small retrospective case series with the inherent limitations, it highlights the diverse patient cohort who can present with bladder perforations. It demonstrates that a high index of suspicion is needed when patients present with abdominal pain and difficulty voiding, often in the absence of a classic history of high impact abdominal trauma. It also highlights that while early surgical repair remains the standard of care for intraperitoneal perforations, conservative measures alone can be successful in select patients. It similarly highlights that some cases of extraperitoneal bladder perforation may require surgical exploration.

## Ethical approval

This study was granted institutional review board exemption.

## Patient consent

The authors confirm that all patients provided both verbal and written consent.
